# Diagnosis and Treatment of Paraneoplastic Neurologic Syndromes

**DOI:** 10.3390/antib12030050

**Published:** 2023-07-31

**Authors:** Daniel Chiu, John Rhee, L. Nicolas Gonzalez Castro

**Affiliations:** 1 Department of Neuro-Oncology, Massachusetts General Hospital Cancer Center, Boston, MA 02114, USA; 2 Center for Neuro-Oncology, Dana-Farber Cancer Institute, Department of Neurology, Brigham and Women’s Hospital, Harvard Medical School, 25 Shattuck Street, Boston, MA 02115, USA

**Keywords:** paraneoplastic neurologic syndrome, onconeural antibodies, autoimmune

## Abstract

Paraneoplastic antibody syndromes result from the anti-tumor antibody response against normal antigens ectopically expressed by tumor cells. Although this antibody response plays an important role in helping clear a nascent or established tumor, the engagement of antigens expressed in healthy tissues can lead to complex clinical syndromes with challenging diagnosis and management. The majority of known paraneoplastic antibody syndromes have been found to affect the central and peripheral nervous system. The present review provides an update on the pathophysiology of paraneoplastic neurologic syndromes, as well as recommendations for their diagnosis and treatment.

## 1. Introduction

### 1.1. Overview and History

Paraneoplastic neurologic syndromes are defined and characterized by an inappropriate immune response targeting native nervous system antigens that are ectopically expressed by a systemic tumor. The first reported case of a possible paraneoplastic neurologic disorder was documented in 1888 by Hermann Oppenheim, a young neurologist working at the Charité Hospital in Berlin [1,2]. He described a case of a 54-year-old female patient presenting with a variety of neurocognitive abnormalities, including agnosia, mood changes and aphasia. She died a few days after her presentation and was found to have a large gastric cancer at autopsy. The careful micro- and macroscopic inspection of the brain, however, did not reveal any pathologic changes to explain the neurologic symptoms. Oppenheim hypothesized “… that the toxic focal neurological symptoms of the brain are in fact caused by the presence of a carcinoma”, thus establishing the idea that cancer can mediate distant neurologic effects through as yet unclear “toxic products”, even in the absence of direct tumor infiltration or neuronal death in the brain [1]. 

Subsequently, the French physician M. Auché described in 1890 the first case of a paraneoplastic syndrome affecting the peripheral nervous system (neuropathy) associated with cancer [3]. This was then followed almost 60 years later by a report from Dr. Derek Denny-Brown describing two cases of neuromyopathy associated with lung cancer [4].

In the 1960s, Drs. Wilkinson and Zeromski at the Western Infirmary in Glasgow described their own cases of patients with neuromyopathy associated with bronchial carcinoma; when they examined the sera of these patients, they found circulating antibodies directed against neurons in a perinuclear pattern [5], thus providing support for Oppenheim’s hypothesis regarding elusive “toxic products” that are able to mediate neurologic effects from a distance. In the 1980s, Graus and colleagues identified and characterized these anti-neuronal nuclear antibodies as anti-Hu [6]. In the following years, additional autoantibodies with characteristic oncologic associations and prototypical neurologic manifestations were identified, thus laying the groundwork for the modern field of paraneoplastic neurologic disorders. Oppenheim’s initial hypothesis regarding the presence of “toxic products’’ proved to be prescient and can at present be understood to represent components of the humoral and cell-mediated immune system (i.e., autoantibodies and cytotoxic T cells) that are generated in the presence of cancer and inappropriately target the nervous system. 

### 1.2. Epidemiology

Paraneoplastic neurologic syndromes (PNSs) are rare, affecting less than 1% of cancer patients overall [7]. The incidence of neurologic paraneoplastic syndromes varies with the specific syndrome and the type of primary tumor. Overrepresented tumors frequently associated with PNSs tend to either: express neuroendocrine proteins (e.g., small-cell lung cancer and neuroblastoma), involve immunoregulatory organs (e.g., thymoma), contain neuronal components (e.g., teratomas), or affect immunoglobulin production (e.g., myeloma) [8]. The most common PNSs are: Lambert–Eaton Myasthenic Syndrome (LEMS), which affects 3.8% of small-cell lung cancer (SCLC) patients [9], and myasthenia gravis (MG), which affects approximately 39% of patients with thymoma [10]. One prospective study found that 9.4% of patients with SCLC have one or more paraneoplastic syndromes, most commonly LEMS, sensory neuropathy, and limbic encephalitis [9]. The incidence of PNSs is much lower (~1%) for other solid tumors [11]. Between 5–15% of patients with plasma cell dyscrasias develop paraneoplastic peripheral neuropathies [12]. The likelihood that a given neurologic disorder can be attributed to a paraneoplastic process varies widely depending on the syndrome, ranging from approximately 60% for LEMS vs. approximately 10% for encephalomyelitis [13].

### 1.3. Pathophysiology

**Figure 1:**
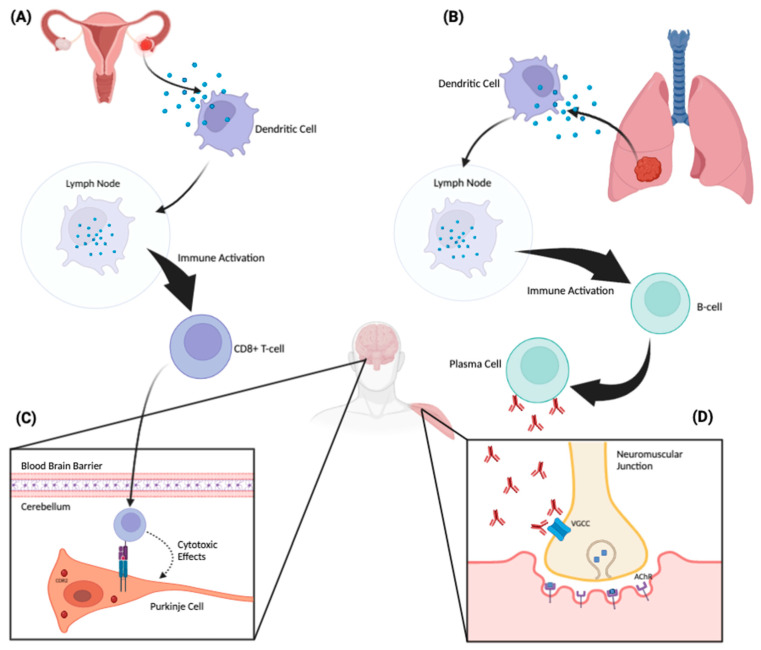
Proposed pathophysiologic mechanism of paraneoplastic neurologic disorders. A tumor aberrantly expresses a neuronal protein (antigen) that the immune system recognizes as non-self. An ovarian tumor (**A**) and a lung tumor (**B**) are depicted as examples. These tumor-associated proteins are phagocytosed by dendritic cells and subsequently presented to lymphocytes in regional lymph nodes. There, they activate antigen-specific CD8+ cytotoxic T cells and antibody-producing B cells. These antibodies and cytotoxic T cells can then trigger an antigen-specific PNS affecting the peripheral or central nervous system. When the corresponding neuronal antigen is located intracellularly, CD8+ cytotoxic T cells can recognize and bind to these antigens when they are presented on the cell surface by MHC-1 molecules (**C**). Through this binding, the T cell can then exert cytotoxic effects on the target neuronal cell. Panel C demonstrates a CDR2-specific T-cell response against a Purkinje cell in the cerebellum. On the other hand, when the corresponding neuronal antigen is located on the cell surface, the antibodies themselves can be pathogenic (**D**). Panel (**D**) depicts antibodies acting directly against voltage-gated calcium channels located on the surface of a presynaptic nerve terminal at the neuromuscular junction. This inhibits the influx of calcium into the nerve, which, in turn, attenuates the release of acetylcholine into the neuromuscular junction, producing the Lambert–Eaton myasthenic syndrome.

PNSs are autoimmune disorders. The pathophysiology of these syndromes is based on an immune response generated against CNS antigens that are normally expressed exclusively in the nervous system, but which are aberrantly and ectopically expressed by tumor cells. The tumor antigen and the neural antigen are identical, but for reasons that are still unclear, the immune system identifies it as foreign and mounts an attack [14]. Tumor-associated intracellular and cell-surface proteins are phagocytosed by dendritic cells and subsequently presented to lymphocytes in regional lymph nodes (Figure 1). There, they activate antigen-specific CD8+ cytotoxic T cells and antibody-producing B cells [14]. The antigen-specific antibodies and cytotoxic T cells that comprise this immune response can then trigger a PNS affecting the peripheral or central nervous system; the latter can be affected if they are able to cross the blood–brain barrier and react with neurons expressing these antigens [14]. From a pathophysiologic standpoint, PNSs can be classified into two groups: (1) PNSs with antibodies directed against intracellular neuronal proteins (the so-called “classical” or “onconeural” proteins) and (2) PNSs with antibodies directed against synaptic or cell membrane proteins. Note that antibodies help to characterize and diagnose the syndrome, but they are not necessarily pathogenic. 

If identified, onconeural antibodies directed against intracellular neuronal proteins are suggestive, though not definitely indicative, of an underlying tumor. These antibodies—also termed “high-risk” because of their frequent association with malignancy—are associated with tumors in >70% of cases [15]. They include the following fully characterized antibodies: anti-Hu (a.k.a., anti-ANNA-1), anti-Ri (a.k.a., anti-ANNA-2), anti-Yo (a.k.a., anti-PCA-1), anti-amphiphysin, anti-Ma2, anti-Tr, anti-CRMP-5 and anti-recoverin antibodies. These antibodies are associated with specific PNSs, but they are not directly pathogenic; neurologic effects and neuronal loss are, instead, mediated by the cytotoxic effects of T lymphocytes, although the antibodies may induce or enhance the T-cell response [16]. On the other hand, PNSs with antibodies directed against synaptic or cell membrane proteins both establish the diagnosis and are also directly pathogenic. Examples include anti-NMDAR, anti-AMPAR, anti-P/Q VGCC, anti-AChR, anti-LGI1, anti-GABA-A, anti-GABA-B, anti-CASPR2, anti-GAD65, anti-GlyR, anti-mGluR1 and anti-mGluR5 antibodies. 

## 2. Approach to Diagnosis

The neurologic symptoms can often precede the discovery of an otherwise occult tumor; in about two thirds of cases, PNSs develop prior to the diagnosis of cancer [17,18]. Most PNSs develop in a subacute manner, with symptoms evolving over weeks to months. The subacute evolution of a characteristic paraneoplastic neurologic syndrome in a patient with cancer should prompt the consideration of an occult malignancy. Similarly, the identification of paraneoplastic antibodies—with or without neurologic symptoms—always warrants an evaluation for malignancy. 

In this paper, we offer a three-step approach to diagnosis (Table 1). First, a thorough clinical and neurologic examination should be undertaken to define the syndrome clinically. Careful consideration should be paid to the patient’s symptoms, signs and their temporal evolution. If the clinical syndrome does not match the description of a known syndrome, the clinician should review the literature for new syndromes. Second, laboratory investigations should be initiated to evaluate for common causes that could account for the symptoms as well as for paraneoplastic causes. The clinician should carefully consider which antibodies would mediate the observed syndrome and support the diagnosis. Appropriate antibody testing in blood and/or CSF should then be undertaken to identify characteristic onconeural antibodies (Table 2).

Of note, approximately 30% of patients with presumed PNS do not have detectable antibodies in either serum or CSF, and thus, the absence of specific antibodies does not necessarily rule out PNS, since these may be antibodies that have not yet been discovered [7]. Third, a review of the diagnostic criteria should be undertaken to delineate a “definite” vs. “possible” diagnosis of PNS; this will guide the need for additional testing and help to define the necessary depth of malignancy screening [19]. 

An international panel of neurologists convened in 2004 to propose two levels of evidence—“definite” or “possible” to define a neurologic syndrome as paraneoplastic, based on the presence/absence of cancer, the presence of classical clinical features, response to anti-cancer treatment and presence of well-characterized onconeural antibodies [19]. These criteria were further refined and updated in 2021 by an expert panel delineating “definite” vs. “probable” vs. “possible” PNS utilizing a combination of clinical phenotype, antibody type, the presence/absence of cancer and time of follow-up (Table 3). The panel further stratified onconeural antibodies into a “high-risk” category (>70% associated with cancer), an “intermediate-risk” category (30–70% associated with cancer), and a “low-risk” category (<30% associated with cancer). According to the new criteria, the diagnosis of definite PNS requires, at present, the presence of high- or intermediate-risk antibodies [15]. 

The evaluation of patients with a suspected PNS should include CSF analysis to search for relevant autoantibodies and to interrogate alternative differential possibilities. CSF autoantibodies are frequently detected in PNS; in certain syndromes, such as anti-NMDAR encephalitis, CSF autoantibodies are highly specific and critical to establishing a diagnosis [20]. Broader CSF markers are frequently abnormal; common findings include: lymphocytic pleocytosis, elevated protein levels and the presence of oligoclonal bands. As a general rule, antibodies against intracellular proteins are usually detected in sera but not CSF, and antibodies against synaptic or cell membrane proteins are sometimes detected in CSF and not in sera. However, sensitivity and specificity for serum vs. CSF analysis vary among different antibodies, and thus it is recommended to perform antibody testing in both samples [15]. 

Neuroimaging is helpful to exclude other non-paraneoplastic disease processes, but it is often normal [21]. In general, MRI has suboptimal specificity for PNSs, save for perhaps limbic encephalitis, which has characteristic T2/FLAIR abnormalities in the mesial temporal lobes [17,22]. FDG-PET-based imaging may show hypermetabolic abnormalities in symptomatic brain regions even when MRI is negative [23]. 

Additional electrophysiologic testing can be undertaken to augment the findings of neuroimaging and CSF studies. Patients presenting with PNS and encephalopathy will often have abnormal electroencephalogram (EEG) findings. One notable and distinctive EEG pattern is the extreme delta brush observed in up to 30% of patients with anti-NMDAR antibody encephalitis, but which is often only apparent in the profoundly encephalopathic patient [24]. EMG/NCS may be helpful in delineating neuromuscular dysfunction in patients with VGKC/CASPR2 autoantibodies in which neuromyotonia is common [25]. 

For over 90% of patients with PNS and solid tumors, the primary tumor will declare itself and be identified within one year of the onset of PNS [21]. However, there are rare instances when the expected tumor is not discovered until several years after the demonstration of neurologic symptoms [26]. In those patients with definite or possible PNS, in whom cancer has not yet been identified, a thorough evaluation for malignancy should be undertaken, focused initially on those tumors most commonly associated with the patient’s syndrome, but expanded if no tumor is initially found, as unexpected cancer–antibody associations may occur. Due to the common association of breast and gynecologic malignancies with PNS, a mammogram and CT or MRI of the pelvis should be undertaken in women who are suspected of having PNS. In patients exhibiting brainstem or limbic encephalitis, an ultrasound or CT/MRI of the pelvis should be undertaken to search for possible testicular tumor (men) or ovarian teratoma (women). Whole-body PET imaging may detect occult tumors that escape detection by other means; a combined multimodal approach with PET and CT imaging has increased sensitivity for the detection of occult cancers [27]. When studies are negative, patients should continue to be evaluated periodically. A 2010 study group recommended imaging patients again between 3 and 6 months after an initial evaluation and continuing with periodic screening every 6 months for up to 4 years [28]. 

## 3. Approach to the Selection of Paraneoplastic Neurologic Syndromes 

In this section, we take a neuroanatomic approach to organizing specific PNSs, based upon the region of the neuraxis affected.

### 3.1. Syndromes Affecting the Brain and Spinal Cord

#### 3.1.1. Paraneoplastic Limbic Encephalitis

Encephalitis involving the limbic structures (amygdala, hippocampi, cingulate gyrus, hypothalami and limbic cortex) typically evolves in a subacute fashion. Clinical features include behavioral disturbances, neuropsychiatric dysfunction, amnesia and seizures [29]. Characteristic MRI findings include unilateral or bilateral T2/FLAIR hyperintensities in the medial temporal lobes involving the hippocampus and amygdala most prominently. Contrast enhancement is rare, and its presence should alert the clinician to the possibility of an alternative diagnosis, such as metastasis [30]. CSF analysis will often reveal a lymphocytic pleocytosis. The most commonly associated cancers are SCLC, testicular germ-cell tumors, ovarian teratomas, thymoma and Hodgkin’s lymphoma [8,29]. The identification of characteristic onconeural antibodies may guide a targeted search for a causative neoplasm. For instance, the presence of anti-Ma2 antibodies is suggestive of a testicular or extra-testicular germ-cell tumor [31]. The presence of anti-Hu antibodies should prompt a search for SCLC [32]. Anti-mGluR5 antibodies are most suggestive of underlying Hodgkin’s lymphoma [33]. Empiric therapy for viral encephalitis is warranted until a viral etiology is excluded.

#### 3.1.2. Paraneoplastic Encephalomyelitis

Paraneoplastic encephalomyelitis (PEM) is a multifocal disorder affecting multiple levels of the neuraxis, including the brain and the spinal cord. Clinical features encompass brainstem encephalitis, cerebellar degeneration, myelitis and/or sensory and autonomic neuropathies. Multifocal syndromes without encephalitis, including myeloneuropathy and paraneoplastic-subacute-combined degeneration, would also fall under this broad category [34]. The most common underlying malignancy is SCLC. Many of these patients will have anti-Hu or anti-CRMP5 antibodies [35]. Unfortunately, PEM is often refractory to treatment with relatively poor long-term prognosis. In one series examining a cohort of patients with PEM harboring anti-Hu antibodies, over half of patients had severe disability at the time of diagnosis, which in turn was associated with a higher mortality [36]. Prompt and early treatment can lead to stabilization.

#### 3.1.3. Paraneoplastic Cerebellar Degeneration

Paraneoplastic cerebellar degeneration (PCD) is characterized clinically by an acute or subacute onset of progressive cerebellar dysfunction, with typical symptoms including dizziness, diplopia, dysarthria and nausea/vomiting. A neurologic exam may reveal truncal, appendicular or gait ataxia as well as downbeating nystagmus [8]. Neuroimaging with MRI may reveal cerebellar atrophy and Purkinje cell degeneration late in the disease course. The most commonly associated tumors are: SCLC, breast cancer, ovarian cancer and Hodgkin’s lymphoma [37]. Typically associated onconeural antibodies include: anti-PCA1 (anti-Yo), anti-PCA-Tr and mGlur-1. Unfortunately, PCD is often refractory to treatment. Treatment with plasma exchange and/or IVIG therapy can lead to symptomatic improvement, although effects may not be durable, and no standardized treatment approach has yet been validated [38]. 

#### 3.1.4. Paraneoplastic Opsoclonus-Myoclonus Syndrome

Paraneoplastic opsoclonus-myoclonus (POM) syndrome is also known as the “dancing eyes, dancing feet” syndrome, due to a clinical presentation characterized by rapid, irregular eye movements (opsoclonus) in association with involuntary jerks of the head, trunk or extremities (myoclonus) [8]. About half of pediatric cases are associated with neuroblastoma [39]. Most adults with POM have an underlying SCLC, but other cancers have been associated, including breast adenocarcinoma, ovarian teratoma and testicular germ-cell tumor. Many patients do not have well-characterized antibodies, save for a small subgroup of patients—usually with breast or gynecologic cancers—who harbor anti-Ri antibodies. Other onconeural antibodies that are less commonly associated with POM include: anti-Hu, Ma2, CRMP5 and NMDAR [40]. The hallmark of therapy is the treatment of the underlying malignancy and early immunosuppressive therapy. Symptomatic treatments can be used as adjuncts and include: clonazepam, levetiracetam, valproic acid, baclofen and gabapentin [41]. 

### 3.2. Syndromes Affecting Spinal Ganglia or Peripheral Nerves

#### 3.2.1. Paraneoplastic Sensory Neuronopathy

This disorder arises due to damage to the neurons within the dorsal root ganglia. Patients develop progressive loss of all sensory modalities and can also have burning pain. Symptoms can progress in an asymmetric fashion, as opposed to the typically symmetric manner in which neuropathies due to toxic/metabolic disturbances present [42]. Upon examination, patients may exhibit an impairment of proprioception, sensory ataxia and diminished or absent deep tendon reflexes. Electrophysiologic evaluation will reveal absent or reduced sensory nerve action potentials with preserved motor responses [43]. The most commonly associated malignancy is SCLC (70–80% of cases) [44]. Common onconeural antibodies associated with this disorder include: anti-Hu, amphiphysin, CRMP5 and PCA2 antibodies [34]. Prompt treatment with corticosteroids may help to improve the sensory deficit [45]. 

#### 3.2.2. Autonomic Neuropathy

Autonomic neuropathies present with characteristic signs/symptoms of orthostatic intolerance, dry mouth, incontinence, constipation and cardiac arrhythmias. SCLC is a common oncologic association. Patients with autonomic neuropathy may harbor anti-Hu, CRMP5 or ganglionic AChR antibodies [46]. 

### 3.3. Syndromes Affecting the Neuromuscular Junction (NMJ)

#### 3.3.1. Myasthenia Gravis (MG)

Myasthenia gravis is the most common disorder of the neuromuscular junction in which autoantibodies attack ACh receptors in the postsynaptic membrane, leading to a characteristic clinical presentation of ptosis, oculobulbar dysfunction, fatigable proximal muscle weakness and dysphagia [47]. MG is most frequently associated with Abs against nicotinic AChRs but is also seen with anti-MuSK Abs, the latter especially in young female patients with orofacial and early respiratory weakness [48]. Increased jitters (variability in conduction time from nerve to muscle) or abnormal repetitive nerve stimulation (>10% decrement) can be observed in EMG/NCS patients. Approximately 10–15% of patients have a thymoma, and up to 70% of patients can have lymphoid follicular hyperplasia [47]. 

#### 3.3.2. Lambert–Eaton Myasthenic Syndrome (LEMS)

The Lambert–Eaton myasthenic syndrome is characterized by progressive proximal limb weakness, leading to difficulty walking, reduced deep tendon reflexes and autonomic dysfunction [48]. It is associated with antibodies acting against presynaptic membrane P/Q-type VGCC. Symptoms tend to improve with activity (as opposed to MG). EMG/NCS shows low-amplitude CMAPs that facilitate after high-frequency nerve stimulation. LEMS is classically associated with SCLC (approximately 42–61% of cases have underlying SCLC) and anti-VGCC antibodies [49]. 

### 3.4. Syndromes Affecting Muscle Tissues

#### Paraneoplastic Myopathy

Immune-mediated necrotizing myopathy is a rare autoimmune myopathy presenting with acute or subacute onset progressive proximal muscle weakness. It is often associated with SRP53 and HMG Co-A reductase antibodies [34]. While statin use is the most common risk factor for an HMG Co-A reductase antibody-mediated myopathic syndrome, a minority of patients also have paraneoplastic associations. There tends to be minimal inflammation on muscle biopsy, unlike other inflammatory myopathies [50]. Novel myositis associated antibodies, such as anti-SAE1, anti-TIF-gamma and anti-NXP2 antibodies, are associated with an elevated risk of malignancy [51]. Common associated malignancies include gastrointestinal adenocarcinomas, lung adenocarcinoma, ovarian adenocarcinoma and thymoma [50]. 

### 3.5. General Approach to Treatment

The first and most important goal of treating PNSs is identifying and treating the underlying malignancy. In one series examining 200 patients with SCLC and anti-Hu antibodies exhibiting paraneoplastic encephalomyelitis, tumor-directed treatment, regardless of whether immunotherapy was administered to treat the encephalomyelitis directly, resulted in 4.5 × greater odds of an improvement or stabilization of PNS [36,52]. Similarly, patients with paraneoplastic peripheral polyneuropathy from multiple myeloma had marked improvement after aggressive treatment with radio-chemotherapy [53].

#### 3.5.1. Immunosuppression

For PNSs mediated by antibodies directed against synaptic or cell membrane proteins (i.e., the antibodies themselves are directly pathogenic), antibody-depleting and immunosuppressive therapy can be quite effective. First-line immunotherapy typically consists of corticosteroids, intravenous immunoglobulin (IVIG) and/or plasmapheresis [54]. For these patients, early treatment may be associated with better outcomes in terms of neurologic symptom burden [17,54]. However, these treatments often fail in later stages of the disease, when a high intrathecal synthesis of antibodies is present that cannot be sufficiently depleted by the aforementioned treatment approaches. For these patients, second-line immunomodulatory treatments, including rituximab and cyclophosphamide, can be effective [49,55]. In one multi-institutional observational study examining patients with anti-NMDAR encephalitis, patients who failed to improve with first-line therapy, but who went on to receive second-line treatment, had >2.5 higher odds of a better functional outcome than the patients who did not receive second-line treatment; these patients also had a lower risk of relapse [54]. 

Unfortunately, PNSs caused by antibodies directed against intracellular antigens and mediated by T cells tend to be poorly responsive to treatment [17]. For these patients, in addition to treating the primary malignancy, early and aggressive immunomodulatory and immunosuppressive treatment with corticosteroids and IVIG provide the best chance of neurologic recovery, likely because neuronal damage is not yet complete. More aggressive cytotoxic (e.g., cyclophosphamide, an alkylating agent that predominantly depletes T cells) or immunosuppressive (e.g., tacrolimus and cyclosporine) agents can also be trialed. In one small prospective series examining 10 patients with PNS but without active malignancy treated with plasma exchange followed by oral cyclophosphamide, 60% showed stability or improvement in the level of disability [45]. As a negative regulator of T-cell function, tacrolimus may be a promising second-line option for intracellular antibody-associated PNSs. In one single-center retrospective study, patients with high-titer anti-HuD, anti-Yo or anti-CRMP5 autoantibodies were treated with tacrolimus and concurrent oral prednisone; some experienced significant and functionally meaningful neurologic improvement, although larger studies are needed to validate these findings [56]. 

#### 3.5.2. Prognosis

Prognosis can vary depending on the specific PNS and the underlying pathophysiology. As mentioned previously, some disorders, such as LEMS and myasthenia gravis, respond well to immunosuppressive therapy and to treatment of the underlying tumor. On the other hand, disorders involving the central nervous system, such as encephalomyelitis or paraneoplastic cerebellar degeneration, typically have a much poorer response to treatment. The underlying pathobiology may underlie these differential outcomes. Diseases of the neuromuscular junction, such as LEMS or myasthenia gravis, do not involve loss of the parent neuron and thus can recover its function once the causal insult (the pathogenic autoantibodies) has been removed, either directly through immunosuppression or indirectly through the treatment of the underlying, autoantibody-generating neoplasm [14]. Conversely, disorders such as paraneoplastic cerebellar degeneration are typically associated with neuronal damage. Because of their subacute temporal course, diagnosis is often delayed, and the affected neurons often die and are lost, thus making recovery impossible and again underscoring the importance of expeditious treatment. 

Intriguingly, patients with PNSs may have a better prognosis compared with patients with identical tumors but without PNSs [14,57,58,59,60]. This superior prognosis is not simply attributable to the earlier diagnosis of cancer than would otherwise have been the case had the patient not developed neurologic symptoms. In the case of SCLC, for instance, the presence of anti-Hu antibodies may predict treatment response and survival. One case series demonstrated a spontaneous resolution of a lung nodule in a patient with paraneoplastic cerebellar degeneration and anti-Hu antibodies, in the absence of any specific tumor-directed treatment [61]. Another series demonstrated a five-fold higher probability of achieving a complete response to cancer treatment in Hu Ab-positive compared to Hu Ab-negative patients [62,63]. In the case of antibody-positive patients, histopathologic analysis has demonstrated heavy inflammatory infiltration within both tumor and neural tissue, suggesting a more robust anti-tumor inflammatory response and potentially explaining superior treatment response and survival [64]. Patients without a tumor tend to have a higher frequency of relapses than those with a tumor [54].

## 4. Conclusions/Areas of Uncertainty

An increasing number of novel antibodies are being reported that remain as-yet not well-characterized. These include entities such as KCTD16 (associated with limbic encephalitis), neuronal intermediate filament (associated with cerebellar syndrome) and ANNA-3 (associated with sensorimotor neuropathy and cerebellar syndrome) [15]. These antibodies have variable frequencies of cancer association, and their exact pathophysiologic relationship with their associated neurologic syndromes remain unclear. Larger studies are needed to further elucidate their clinical and oncologic significance. 

The utility of assessing antibody titers as a biomarker of response to treatment remains unclear and unvalidated. In one trial evaluating 17 patients with paraneoplastic encephalomyelitis/sensory neuropathy (PEM/SN) with anti-Hu Abs (*n* = 10) or cerebellar degeneration (PCD) with anti-Yo Abs (*n* = 7), treatment with IVIG, methylprednisolone and cyclophosphamide demonstrated a significant decrease in antibody titers in six of seven evaluable patient but with no associated clinical or survival benefit [65]. In another study of nine patients with anti-Hu- or anti-Yo-associated PNS treated with rituximab, no relationship was seen between changes in antibody titers and clinical response [37]. Similarly, another study examined plasma exchange plus either cyclophosphamide or conventional chemotherapy in patients with PNS; amongst the 12 patients who were initially seropositive, 8 had decreased serum antibody titers after treatment (with 5 of 8 clinically improved and 3 of 8 worsened). A total of 2 of the 12 patients had stable antibody titers (both showed clinical improvement after treatment). Additionally, the final two patients showed an increase in antibody titers (with both demonstrating neurologic worsening) [45]. On the other hand, in one study of patients with anti-NMDAR encephalitis, clinical improvement was associated with a decrease in serum antibody titers, whereas patients who failed to improve maintained persistently high antibody titers in both CSF and serum [55]. In another study examining 250 patients with anti-NMDAR encephalitis, CSF and serum titers were noted to be higher in patients with poor clinical outcome than in those with a good outcome [66]. However, the same study also noted that antibody titers tended to decrease over time, regardless of outcome, and that 24 of 28 CSF samples and 17 of 23 serum samples from patients remained antibody-positive even after recovery. 

The exact pathophysiologic relationship of PNS and their associated onconeural antibodies varies depending on the specific syndrome and underlying malignancy. We know that onconeural antibodies are often not directly pathogenic, and that neuronal damage is frequently mediated, instead, by a cytotoxic T-cell response [56]. Thus, antibody-depleting therapies that act primarily on the humoral immune system may not address the underlying pathophysiologic mechanism of neurologic injury, even as they depress the levels of circulating autoantibodies. Even for those antibodies that do play a direct pathogenic role, traditional antibody depleting therapies, such as IVIG and plasma exchange, may fail to adequately remove the antibody from the CNS [67]. The extent to which autoantibody titers can be a reliable marker and correlate of treatment effect is likely dependent on the underlying pathobiology and whether the antibodies themselves are directly pathogenic. More studies are needed to further elucidate their potential utility as a biomarker. 

Immune suppression remains the mainstay of PNS treatment, but more prospective studies are needed to evaluate the efficacy of various immunotherapeutic approaches, with specific attention to their differential effects on syndromes in which the target antigen is intracellular (with a presumed primary role for T cells in the pathophysiology) vs. syndromes in which the target antigen is extracellular/intramembranous. Future efforts might include multimodal approaches that combine antibody-depleting therapies with agents that target other components of cellular immunity, such as T-cell inhibitors. 

## Figures and Tables

**Table 1:** General approach to the diagnosis of paraneoplastic neurologic syndromes.

Use the History and Neurologic Exam to Define the Syndrome and Clinical Phenotype
Perform targeted antibody testing (in both blood and CSF).
Evaluate for systemic malignancy. Employ additional adjunctive testing, as needed (e.g., MRI, EEG and EMG/NCS).

**Table 2:** Antibodies with associated antigens, oncologic associations and prototypical clinical manifestations.

Antibody	Antigen	Antigen Type	Associated Cancer	Syndrome(s)
Anti-Hu (ANNA-1)	HuD and related nuclear proteins	Intracellular	SCLC	Encephalitis, myelitis, encephalomyelitis, sensory neuronopathy, peripheral neuropathy
Anti-Yo (PCA-1)	CDR2	Intracellular	Ovarian, breast	Cerebellar degeneration
Anti-Ri (ANNA-2)	NOVA proteins	Intracellular	Breast, ovarian, SCLC	Cerebellar ataxia, opsoclonus, brainstem encephalitis
Anti-Tr (DNER)	DNER	Intracellular	Hodgkin lymphoma	Cerebellar degeneration
Anti-CV2/CRMP5	CRMP5	Intracellular	SCLC	Encephalitis, myelitis, encephalomyelitis, cerebellar degeneration, optic and peripheral neuropathy
Anti-Ma1, Anti-Ma2 (Ta)	PNMA1, PNMA2	Intracellular	Testicular germ cell tumors	Limbic encephalitis, brainstem encephalitis, cerebellar degeneration
Anti-Recoverin	Recoverin	Intracellular	SCLC, gynecologic cancer	Retinopathy
Anti-Hu2 (ANNA-1); Anti-Ri (ANNA-2); Others	Various	Intracellular	Neuroblastoma	Opsoclonus-myoclonus syndrome (most common pediatric paraneoplastic syndrome)
Anti-GAD65	GAD65 (enzyme that synthesizes GABA)	Intracellular	Usually none	Cerebellar degeneration, Stiff person syndrome
Anti-Amphiphysin	Amphiphysin (synaptic antigen)	Intracellular	Breast, SCLC	Stiff person syndrome, encephalomyelitis
Anti-Caspr2	Caspr2	Intracellular	Thymoma	Neuromyotonia, encephalitis, Morvan syndrome (neuromyotonia + insomnia)
Anti-LGI1	LGI1	Intracellular	Usually none	Faciobrachial dystonic seizures, encephalitis, myoclonus
Anti-NMDAR	NMDAR (ionotropic Glu receptor)	Extracellular	Ovarian teratoma, testicular germ cell tumors	Limbic encephalitis
Anti-AMPA	AMPA (ionotropic Glu receptor)	Extracellular	Lung, breast, thymus	Limbic encephalitis
Anti-GABA-A	GABA-A (ionotropic inhibitory receptor)	Extracellular	Hodgkin lymphoma	Refractory status epilepticus
Anti-GABA-B	GABA-B (metabotropic inhibitory receptor)	Extracellular	SCLC	Limbic encephalitis with seizures, opsoclonus, ataxia
Anti-mGluR1	mGluR1 (cerebellar metabotropic GluR)	Extracellular	Hodgkin lymphoma, prostate	Cerebellar degeneration
Anti-VGCC	VGCC at NMJ	Extracellular	SCLC	LEMS, cerebellar degeneration
Anti-AChR	AChR at NMJ	Extracellular	Thymoma	Myasthenia gravis, autonomic neuropathy

**Table 3:** Criteria for definite vs. probable vs. possible PNS (adapted from [15]).

Criteria	Points
Clinical Phenotype Risk Level	
High-risk phenotype (syndrome often triggered by cancer)	3
Intermediate-risk phenotype (can occur with or without cancer)	2
Low-risk phenotype (weaker association with cancer)	1
Laboratory level	
High-risk antibody (>70% cancer association)	3
Intermediate-risk antibody (30–70% cancer association)	2
Low-risk antibody (<30% cancer association)	0
Cancer	
Found, consistent with phenotype and antibody	4
Not found or not consistent with phenotype, with follow-up <2 years	1
Not found, and follow up ≥2 years	0
Score	Diagnostic Level of Confidence
≥8	Definite PNS
6–7	Probable PNS
4–5	Possible PNS
<4	Not PNS

## Data Availability

No new data were created or analyzed in this study. Data sharing is not applicable to this article.
